# Dispersion of oxides, heavy metals, and natural radionuclides in phosphogypsum stockpiles of the phosphate industries in Türkiye

**DOI:** 10.1007/s11356-025-36180-2

**Published:** 2025-03-05

**Authors:** Şeref Turhan, Ergin Murat Altuner, Aytaç Ayata, Ferhat Gezer, Aybaba Hançerlioğulları, Aslı Kurnaz, Muhammet Karataşlı

**Affiliations:** 1 Department of Physics, Faculty of Science, Kastamonu University, 37150 Kastamonu, Türkiye; 2 Department of Biology, Faculty of Science, Kastamonu University, 37150 Kastamonu, Türkiye; 3 Department of Physics, Institute of Science, Kastamonu University, 37150 Kastamonu, Türkiye; 4 Adana Metropolitan Municipality Reşatbey Mah. Atatürk Cad. Seyhan, Adana, Türkiye; 5 Department of Basic Sciences, Faculty of Engineering, Architecture and Design, Kahramanmaraş İstiklal University, 46100 Kahramanmaraş, Türkiye

**Keywords:** Phosphogypsum; Major and minor oxide; Heavy metal; Naturally occurring radionuclides, EDXRF

## Abstract

Heavy metal (HM) and naturally occurring radioactive materials (NORM) pollution is continuously increasing due to anthropogenic activities, and it is one of the global problems that pose a threat to human and environmental health. Phosphogypsum (PG), a by-product of phosphate fertilizer (PF) production, is an industrial solid waste produced in large quantities worldwide, and much of it is stockpiled. PG contains enhanced levels of toxic substances such as HMs and NORMs. The first detailed study on the determination of major-minor oxides, HMs, and NORMs contents of PGs collected from waste stockpiles at phosphate fertilizer factories (PFFs) in Türkiye was carried out using energy-dispersive X-ray fluorescence spectrometry to obtain data for the reuse of these wastes and a better characterization of the waste deposit. The major-minor oxides analyzed in the PG samples were ranked as SO_3_ (53.22%) > CaO (36.84%) > SiO_2_ (2.08%) > P_2_O_5_ (1.21%) > Al_2_O_3_ (0.13%) > Fe_2_O_3_ (0.11%) according to their average contents. The average contents (mg/kg) of HMs and NORMs analyzed in the PG samples were listed as follows: Fe (744.2) > Ti (102.1) > Zn (59.2) > Mn (28.9) > Ni (18.4) > Cr (15.2) > Co (14.6) > Cu (13.5) > Zr (10.8) > Pb (8.8) > Cd (8.5) > V (7.4) > U (4.9) > Th (4.2). The results revealed that HMs, except Cd and U, were lower than the Earth’s crust averages and maximum soil contaminant levels recommended in the Turkish Regulation on Control of Soil Pollution.

**Supplementary Information:**

The online version contains supplementary material available at 10.1007/s11356-025-36180-2.

## Introduction

Pollution of heavy metals (HMs) and natural or artificial radionuclides is one of the well-known global environmental problems that pose serious dangers to human life and ecosystems (Fashola et al. 2016; Gautam and Aithekar 2019; Ali et al. 2019; Kaur et al. 2021; Tan et al. 2023; Chandra et al. 2023). Although natural processes (volcanic eruptions, soil erosion, geological weathering, metal corrosion, etc.) increase HM pollution, the important cause of pollution, which reaches levels that threaten human and environmental health, is anthropogenic activities such as mining operations, which are the prime source of pollution, foundries, smelting, fertilizers, pesticides, waste dumps, etc. (Ali et al. 2019; Briffa et al. 2020; Saraswat et al. 2020; Ohiagu et al. 2022). HMs, which have persistent, toxic, and non-biodegradable properties, are heterogeneous elements that vary depending on their chemical properties and biological functions (Ali et al. 2019). However, according to their roles in biological systems, HMs can be evaluated as essential and non-essential. Trace essential HMs such as Fe, Zn, Cu, Mn, and Cr (III) have biological importance in small amounts to maintain the health and normal functioning of the human body and also are micronutrients for plants depending on the dose and duration of exposure (Ali et al. 2019; Tchounwou et al. 2012). Trace non-essential HMs such as Cd, Hg, As, Pb, and Cr (VI) have no biological role and can be toxic even in a certain form and at quite low concentrations (Ali et al. 2019; Tchounwou et al. 2012). Radionuclides that emit ionizing radiations (alpha-, beta-, X- and gamma-rays) can be classified as natural and artificial (anthropogenic) radionuclides (Tan et al. 2023). The International Agency for Research on Cancer (IARC) considers all types of ionizing radiation to be harmful to human health (Chandra et al. 2023). The most abundant naturally occurring radioactive materials (NORMs) in the Earth’s crust and soils contain uranium, thorium, radium, and radio-potassium (Tan et al. 2023; Chandra et al. 2023). Besides, various anthropogenic sources such as the disposal of radioactive waste, the mining industry, the production and processing of phosphate fertilizers (PFs), and the production of electricity from fossil fuels affect the distribution of NORMs (Chandra et al. 2023). When substantial amounts of NORMs are released into the environment from these anthropogenic sources, they eventually seep into soil, water, and plants. These NORMs are the primary source of human exposure to ionizing radiation and can potentially cause unfavorable health effects (Machraoui et al. 2024).

Phosphorus, which is one of the basic nutrients required for the healthier, more efficient growth and development of plant reproductive organs, is supplied from PF, which is a mineral fertilizer whose raw material is phosphoric acid (H_3_PO_4_). Phosphoric acid is produced from phosphate ores, which are usually obtained from sedimentary marine and magmatic phosphate deposits, generally using wet and thermal processes (IAEA 2013; Akfas et al. 2024). As a result of the wet process, in addition to phosphoric acid, phosphogypsum (PG, CaSO_4_) is also obtained as a by-product or waste (Noli et al. 2023; Ennaciri and Bettach 2024). Approximately 300 million tons of PG is produced globally each year (Saadaoui et al. 2017; Noli et al. 2023; Ennaciri and Bettach 2024). About 14–15% of these PG productions are recycled, while the remaining is stockpiled into landfills or mounds known as gypsum piles or gyp-stacks or is discharged into the sea surface, surface waters without any prior treatment (Muhanbet et al. 2016; Rashad 2017; Noli et al. 2023; Ennaciri and Bettach 2024). PG is a harmful and toxic industrial waste because it contains impurities such as residual acids, soluble phosphorus, soluble fluorine, sulfate, HMs, and NORMs posing a serious threat to the environment, especially in air, soil, and water resources near gyp stacks (Santos et al. 2002; Tayibi et al. 2009; Muhanbet et al. 2016; Noli et al. 2023; Yan et al. 2024; Ennaciri and Bettach 2024). Therefore, having knowledge related to the chemical composition and the amount and general behavior of impurities of PG waste is critical for making an informed decision regarding treatment and recovery strategies, ensuring the safety of waste management and evaluating its operational effectiveness, allowing PG stockpiles to remain in situ with reasonable remediation (Akfas et al. 2024; Ennaciri and Bettach 2024).

In the literature, there are many publications on the determination of major, minor, and trace elements and natural radionuclide contents of PG wastes obtained from many countries (Burnett and Elzerman 2001; Al-Masri et al. 2004; Fávaro 2005; Papastefanou et al. 2006; Ajam et al. 2009; El Afifi et al. 2009; Roselli et al. 2009; Villalobos et al. 2010; Zielinski et al. 2011; Ali and Awad 2015; Olszewski et al. 2016; Bisone et al. 2017; Madruga et al. 2019; Ndour et al. 2021; Bouargane et al. 2023; Yan et al. 2024). However, although a few studies were conducted on the determination of activity concentrations of natural radionuclides contained in some PG wastes in Türkiye, according to our literature search, there is a lack of data on the chemical composition (major, minor, trace elements and radionuclides) of PG wastes throughout Türkiye. This study aims to contribute to the knowledge about chemical and radionuclide distributions of long-term stored PG piles in PF production plants in Türkiye. In this study, six major-minor oxides, 12 heavy metals, and two naturally occurring radionuclides contained in PG samples collected from PF plants were analyzed by energy-dispersive X-ray fluorescence (ED-XRF) spectroscopy, and the results were compared with those reported from other countries. The novelty of this study is that it provides guiding information that can be evaluated for safer waste management scenarios of PGs in Türkiye and the use of these wastes in different sectors.

## Materials and methods

### Sampling

**Fig. 1:**
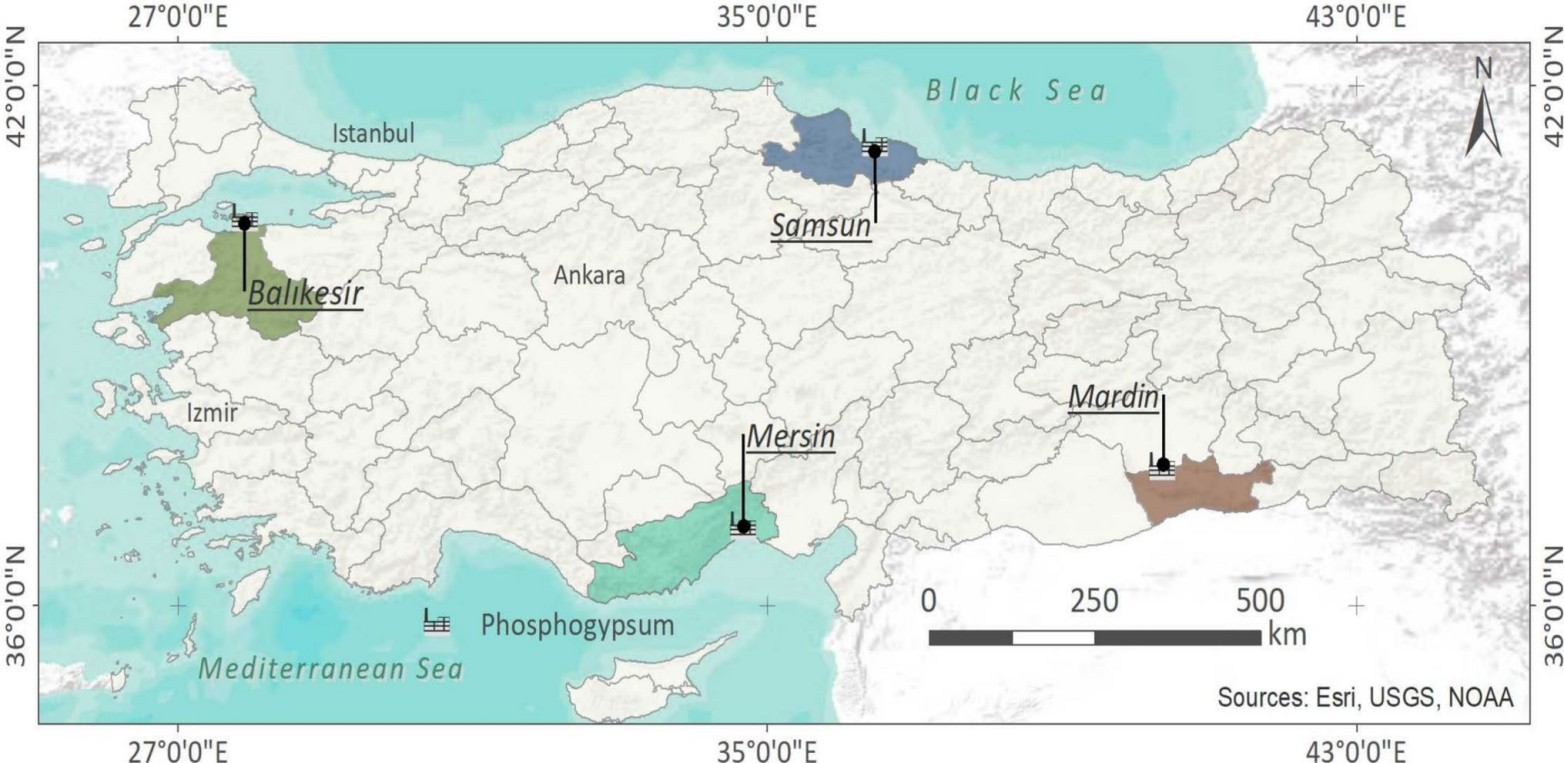
Location of phosphate fertilizer factories in Türkiye

In Türkiye, PFs are produced in phosphate fertilizer factories (PFFs) in Balıkesir (Bandırma), Mardin, Mersin, and Samsun provinces. Phosphate rocks, except for the Mardin PFF, are imported from Egypt, Jordan, and Tunisia. The phosphate rocks used in Mardin PFF are mined in the Mazıdağı district of Mardin province in the Southeastern Anatolia Region of Türkiye. In all PFFs, phosphate rock is converted into phosphoric acid using the wet chemical method. Phosphate rocks used to obtain phosphoric acid in Türkiye are of sedimentary origin. Approximately 3 million tons of PG by-products per year are generated in these PFFs (Akfas et al. 2024). PGs obtained in the form of sludge are usually transported to the landfilling areas near the PFFs, sea, and lakes for long-term storage in the form of piles without processing and recycling and after filtering. Sixty-one PG samples were collected randomly from different points at depths varying from 1 to 10 cm in a gyp-stack (stockpile) of four PFFs, as depicted on the map in Fig. 1, and brought to the sample preparation laboratory in polyethylene packages. Fertilizer factories located in Mardin, Balıkesir, Mersin, and Samsun provinces were coded as PFF1, PFF2, PFF3, and PFF4, respectively. Each sample was then dried in an oven at 110 °C and ground into powder to suit the powder geometry used in the analysis process. Three or five grams of each sample was taken for analysis after homogenization (Turhan et al. 2024).

### Elemental analysis

Elemental analysis of the PG samples was performed by ED-XRF spectrometry (Spectro Xepos, Ametek). The specifications of the ED-XRF spectrometry are presented in Table S1. The ED-XRF spectrometry has software to analyze major, minor, and trace elements among sodium and uranium in environmental samples simultaneously, and it utilizes “non-standard” calibration techniques (Altıkulaç and Turhan 2023; Turhan et al. 2024). The system does not require the calibration of certified reference samples. The user simply supplies relevant initial samples to build the database; the software examines the measured spectra and creates a fingerprint for each sample. After that, the fingerprint of each newly introduced sample can be quickly taken and compared with the rest of the database. Easy validation is achieved by comparing all samples of the database with each other instead of verifying every calibration for every element in every matrix (Spectro Amatek 2024). The spectrum of each PG sample was evaluated using TurboQuant II software. The overall uncertainty (%) of major-minor oxides and HMs were SO_3_ (0.1), CaO (0.6), SiO_2_ (0.3), P_2_O_5_ (0.2), Al_2_O_3_ (5.2), and Fe_2_O_3_ (0.9), Ti (3.0), V (10.6), Cr (4.7), Mn (4.2), Fe (0.7), Co (11.8), Ni (5.1), Cu (7.5), Zn (3.4), Zr (10.2), Cd (10.8), Th (9.4), and U (7.4).

### Statistical analysis

Statistical analysis was performed to evaluate data distribution, assess variance homogeneity, and identify potential differences. The Shapiro–Wilk test was used to check for normality, while the Bartlett test assessed the homogeneity of variances. Results indicated that most data did not follow a normal distribution or exhibit homogeneity of variances. Consequently, a logarithmic transformation was applied, but the transformed data still failed to meet the assumptions of normality and homogeneity.

As a result, the Kruskal–Wallis test (*p* = 0.05) was employed to examine differences among groups, and pairwise Wilcoxon rank-sum tests were conducted to identify significant differences. Additionally, Pearson correlation coefficients were calculated to investigate correlations between HM and NORM concentrations and major and minor oxides in the samples. All statistical analyses were performed using RStudio version 2023.06 (R Core Team R 2023).

### Spatial distribution analysis

The spatial distribution of HM and NORM concentrations was analyzed using QGIS Desktop 3.36.0. The geographic coordinates of each sampling location were mapped, and inverse distance weighting (IDW) interpolation was applied to present the concentrations of HMs and NORMs across the collection sites.

## Results and discussion

### Major and minor oxide composition

**Fig. 2:**
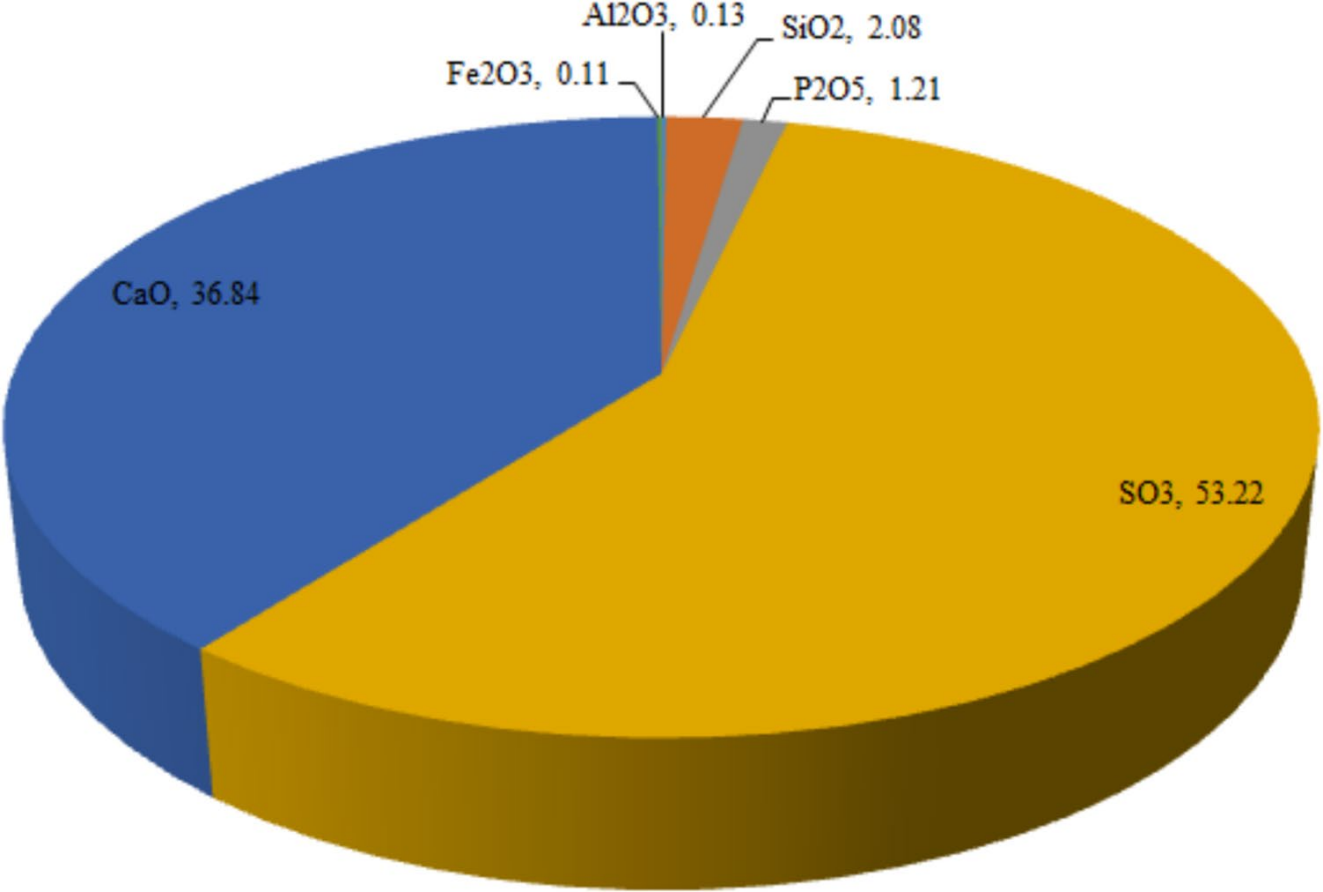
Major and mineral oxides of Turkish phosphogypsum in percentage

The distribution of major (SO_3_, CaO, SiO_2_, and P_2_O_5_) and minor (Al_2_O_3_ and Fe_2_O_3_) oxides analyzed in all studied PG samples is shown in Fig. 2. The average and range (minimum–maximum) values of major and minor oxide contents of each PFF PG wastes are presented in Table 1. Table 2 shows the oxide composition of Turkish PG compared to those reported from other countries. From Fig. 2, the oxides were ranked in decreasing order as SO_3_ (53.22%) > CaO (36.84%) > SiO_2_ (2.08%) > P_2_O_5_ (1.21%) > Al_2_O_3_ (0.13%) > Fe_2_O_3_ (0.11%). The contents (in dry weight) of SO_3_, CaO, SiO_2_, P_2_O_5_, Al_2_O_3_, and Fe_2_O_3_ in the samples varied from 37.19 to 65.89%, 29.81 to 41.63%, 0.13 to 5.31%, 0.38 to 3.65%, 0.01 to 1.25%, and 0.01 to 0.82%, respectively. From Table 1, the highest contents of Al_2_O_3_, SiO_2_, CaO, and Fe_2_O_3_ were analyzed in PG samples supplied from the factory-coded PFF4, while the highest contents of SO_3_ and P_2_O_5_ were analyzed in PG samples from the PFF2 and PFF4, respectively. The oxides in PG samples from PFFs were ranked in decreasing order as Fe_2_O_3_ < Al_2_O_3_ < P_2_O_5_ < SiO_2_ < CaO < SO_3_ for PFF1, PFF2, and PFF4, and Fe_2_O_3_ < Al_2_O_3_ < SiO_2_ < P_2_O_5_ < CaO < SO_3_ for PFF3. As can be seen from Table 2, the oxide composition of Turkish PG is consistent with those of PG produced from different sedimentary sources.

**Table 1:** Contents of oxides in PG samples from phosphate fertilizer factories in Türkiye

Fabric code		Oxide content (%, dry weight)					
		Al_2_O_3_	SiO2	P_2_O_5_	SO_3_	CaO	Fe_2_O_3_
PFF1	Average	0.06	3.58	1.98	51.55	35.02	0.04
	Range	0.01–0.20	0.60–5.27	0.53–3.65	37.92–62.08	29.81–38.25	0.01–0.17
PFF2	Average	0.04	0.82	0.63	59.24	35.97	0.03
	Range	0.02–0.09	0.42–1.10	0.38–0.74	37.19–65.89	33.73–38.13	0.02–0.04
PFF3	Average	0.04	0.18	0.92	49.62	38.00	0.02
	Range	0.02–0.08	0.13–0.23	0.81–1.25	45.82–63.96	36.44–39.98	0.02–0.03
PFF4	Average	0.26	2.11	0.83	54.23	38.52	0.26
	Range	0.04–1.25	0.72–5.31	0.63–1.24	45.42–65.37	33.45–41.63	0.02–0.82

**Table 2:** Comparison of oxide contents in Turkish PG with those in PG samples in different countries

Country	CaO	SiO_2_	Al_2_O_3_	Fe_2_O_3_	SO_3_	P_2_O_5_	Reference
ABD	25–31	3–18	0.1–0.3	0.2	55–58	0.5–4.0	Tayibi et al. (2009)
Algeria	31.2	0.9	0.1	0.03	40.9	0.87	Kacimi et al. (2006)
China	34.5	3.2	1.1	0.3	47.3	4.2	Yan et al. (2024)
Tunisia	32.8–37.2	1.4–1.0	0.06–0.1	0.03–.0.1	37.5–44.0	1.1–1.7	Akfas et al. (2024)
Jordan	31.8–35.5	9.2–11.8	0.3–0.5	0.15–0.2	39.8–51.2	1.8–4.7	Akfas et al. (2024)
India	31.1	0.3	0.5*		43.2	0.5	Manjit and Mridul (2005)
Morocco	32.2–35.0	1.4–1.8	0.2–0.5	–	29.1–44.3	–	Akfas et al. (2024)
Egypt	28.3–38.4	5.5–8.3	0.17–0.2	–	40.4–53.2	1.98–2.1	Akfas et al. (2024)
Spain	37.0–38.1	–	0.18–0.19	0.2–0.3	48.1–51.5	0.3–0.7	Akfas et al. (2024)
Several countries	24–34	0.5–18	0.05–0.6	0.01–0.25	48–58	0.05–8	IAEA (2013)
Türkiye	36.8	2.1	0.13	0.11	53.2	1.2	This study

^*^A1_2_O_3_ + Fe_2_O_3_

### Heavy metal and radionuclide composition

**Fig. 3:**
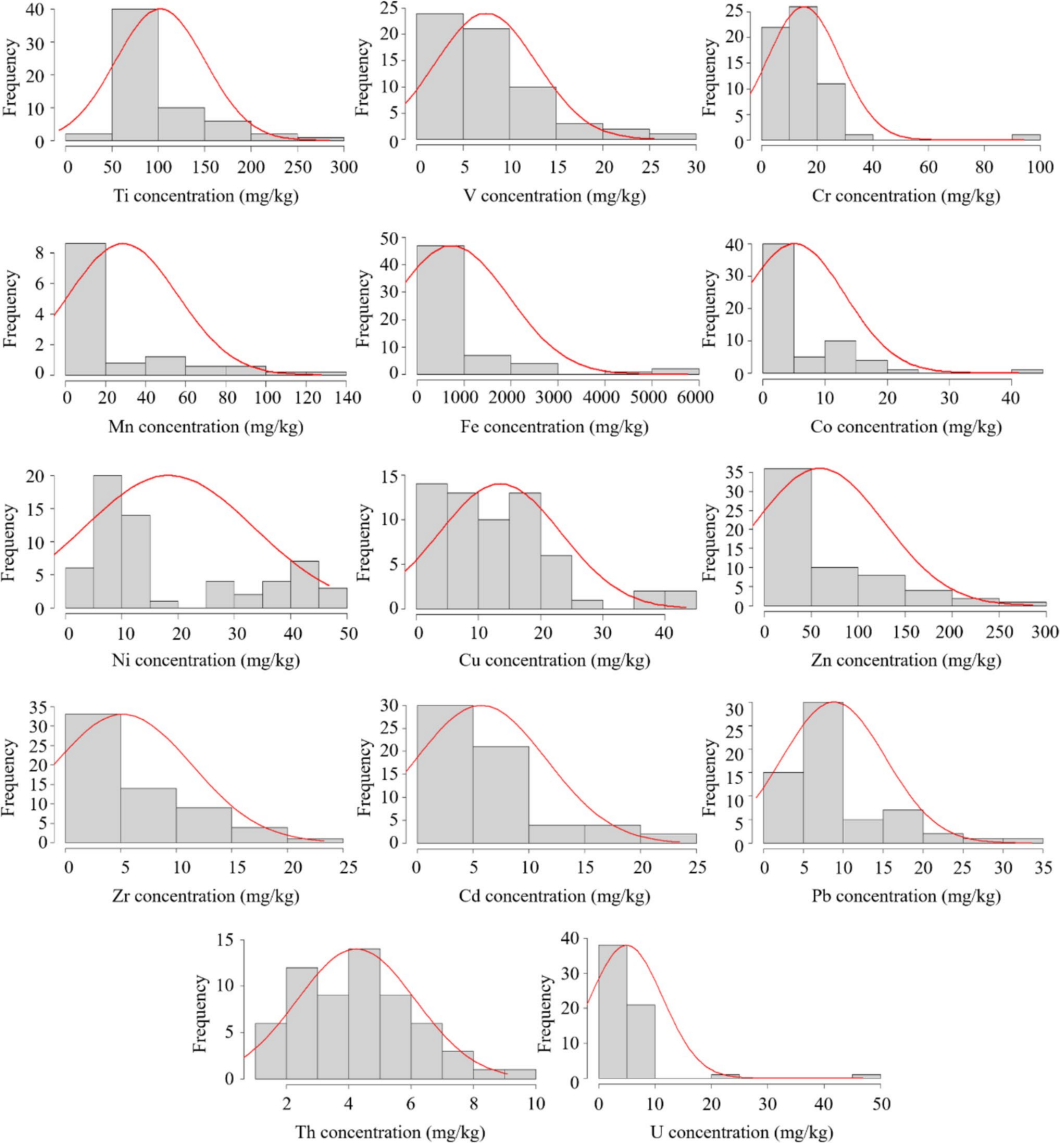
Frequency distributions of HMs and NORMs contents in PG samples

The contents of As and Hg in all PG samples were found below the detection limit of 0.5 and 1.0 mg/kg, respectively. Some descriptive statistical data of the contents of HMs and NORMs analyzed in all PG samples is given in Table 3. The frequency distribution of the content of HMs and NORMs is shown in Fig. 3. The average and range (minimum–maximum) values of HMs and NORMs contents of each PFF PG waste in Türkiye and several countries are given in Table 4.

**Table 3:** Some descriptive statistical data on the content of HMs and NORMs in PG samples

HM and NORM	Average	Median	SD*	SE*	Kurtosis	Skewness	Min	Max	*N*
Ti	102.1	85.1	48.3	6.2	3.0	1.7	42.8	284.7	61
V	7.4	6.1	5.5	0.7	2.2	1.4	1.1	25.5	61
Cr	15.2	15.4	13.1	1.7	21.3	3.6	1.4	94.1	61
Mn	28.9	16.3	27.4	3.5	3.7	2.0	9.4	127.7	61
Fe	744.2	242.0	1204.4	154.2	8.3	2.9	84.2	5760.0	61
Co	14.6	12.3	7.2	0.9	9.2	2.8	9.6	41.1	21
Ni	18.4	11.6	15.1	1.9	− 1.0	0.8	1.4	46.9	61
Cu	13.5	11.3	9.8	1.3	1.6	1.3	2.4	43.4	61
Zn	59.2	33.7	68.6	8.8	1.8	1.5	3.0	285.9	61
Zr	10.8	9.3	4.5	0.6	1.1	1.1	3.5	23.3	29
Cd	8.5	6.9	5.3	0.7	1.4	1.4	2.9	23.5	41
Pb	8.8	5.8	6.5	0.8	4.2	2.1	4.1	33.6	61
Th	4.2	4.4	1.9	0.2	− 0.5	0.3	1.1	9.1	61
U	4.9	3.4	6.4	0.8	31.1	5.0	1.1	46.9	61

*SD* standard deviation, *SE* standard error

**Table 4:** Contents of HMs and NORMs in PG samples from phosphate fertilizer factories in Türkiye

HM and NORM	Fabric code								PG in several countries
	PFF1		PFF2		PFF3		PFF4		
	Content (mg/kg, dry weight)								
	Average	Range	Average	Range	Average	Range	Average	Range	Range*
Ti	77.2	60.9–195.4	111.2	77.2–174.9	86.7	71.2–97.3	132.7	42.8–284.7	26–470
V	3.3	1.1–10.6	6.6	4.6–12.5	10.8	4.6–25.5	10.4	1.6–24.2	0.7–880
Cr	19.7	12.9–33.8	2.8	1.4–4.4	6.9	2.1–14.9	20.5	4.6–94.1	1.6–670
Mn	14.4	11.6–18	14.4	9.4–39.3	16.5	14.5–18.2	57.3	11.2–127.7	3.5–20.0
Fe	267.1	84.2–1191	205.0	155.1–263.5	164.7	135.2–200.3	1806.5	151.4–5760	128–43,071
Co	14.6	9.6–41.1	< 2.0		< 2.0		< 2.0		0.1–17.4
Ni	11.3	4.9–15.1	6.0	1.4–8	5.2	2.3–7.6	38.7	27.4–46.9	1.7–250
Cu	15.2	4.7–23.7	3.8	2.4–5.5	5.7	3.7–8	20.4	3.8–43.4	1.6–195
Zn	42.4	6.4–84.7	5.3	4.2–6.6	4.1	3–5.6	131.3	7.9–285.9	1.6–315
Zr	< 1.0		9.4	6.1–19.3	< 1.0		11.4	3.5–23.3	10–110
Cd	6.5	2.9–12.8	4.6	2.8–6.8	< 2.0		10.8	2.9–23.5	0.13–40
Pb	5.1	4.1–7.6	6.4	4.8–8.8	5.5	4.7–6.4	15.6	4.4–33.6	0.8–23.3
Th	2.4	1.1–9.1	7.0	6.3–8.2	5.2	4.6–6.4	4.3	3.3–5.9	0.2–43.7
U	2.1	1.1–4.8	1.5	1.1–1.9	7.1	5.2–8.2	8.3	2.4–46.9	0.6–27.5

^*^Values analyzed in PG samples from various countries (Santos et al. 2002; IAEA 2013; Saadaoui et al. 2017; Noli et al. 2023; Akfas et al. 2024)

**Fig. 4:**
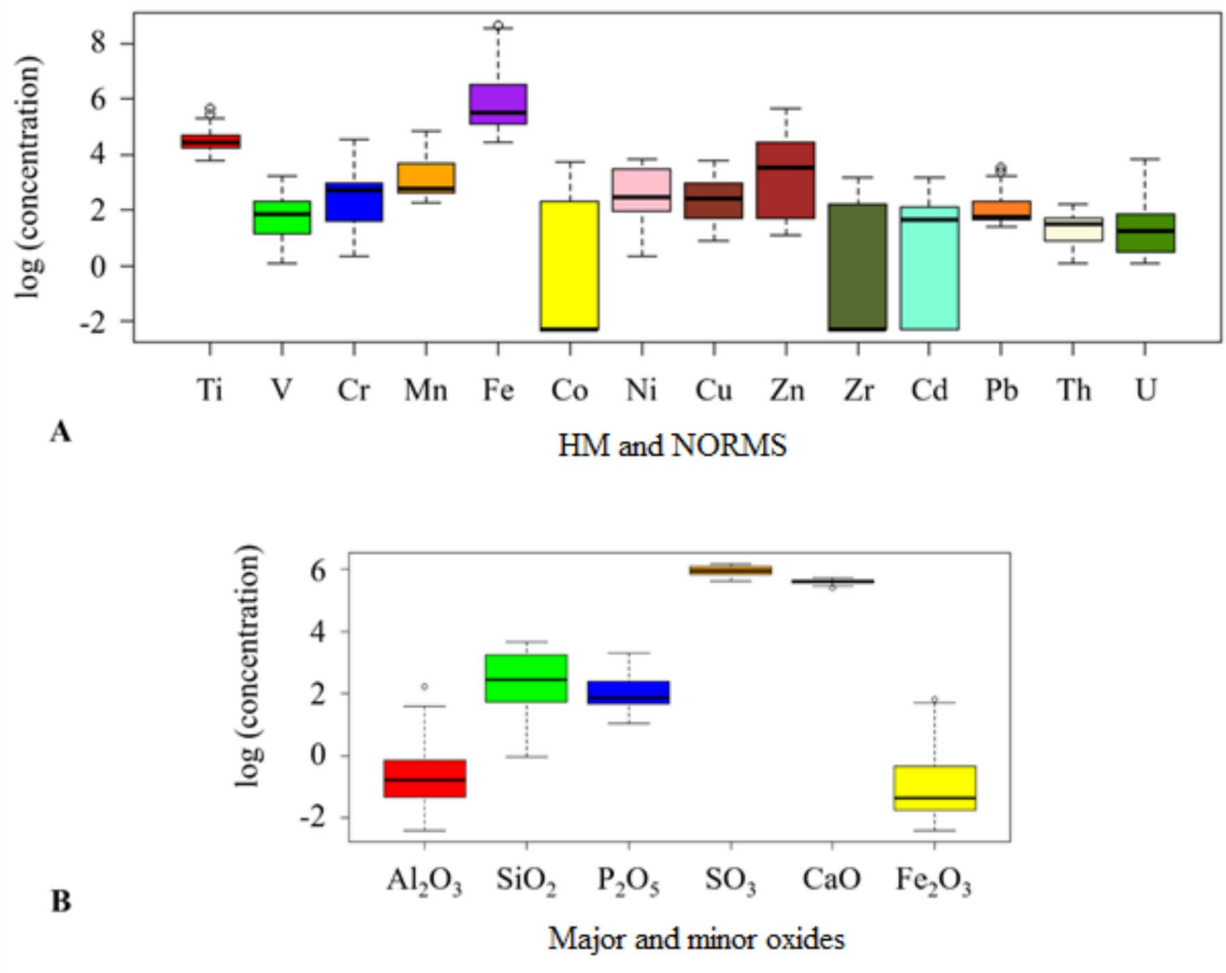
The box plot for the logarithmic transformed data. **a** HM and NORMs. **b** Major and minor oxides

From Table 3, HMs and NORMs analyzed in the PG samples were ranked in decreasing order according to their average contents (in mg/kg) as Fe (744.2) > Ti (102.1) > Zn (59.2) > Mn (28.9) > Ni (18.4) > Cr (15.2) > Co (14.6) > Cu (13.5) > Zr (10.8) > Pb (8.8) > Cd (8.5) > V (7.4) > U (4.9) > Th (4.2). The box plot for the concentration distribution of HM and NORMs for logarithmically transformed data is given in Fig. 4. The contents of Fe, Ti, Zn, Mn, Ni, Cr, Co, Cu, Zr, Pb, Cd, and V in PG samples varied from 84.2 to 5760.0, 42.8 to 284.7, 3.0 to 285.9, 9.4 to 127.7, 1.4 to 46.9, 1.4 to 94.1, < 2.0 to 41.1, 2.4 to 43.4, < 1.0 to 23.3, 4.1 to 33.6, < 2.0 to 23.5, and 1.1 to 25.5 mg/kg, respectively. The average content of Cd analyzed in PG is significantly greater than the Earth’s crust average of 0.13 mg/kg and the maximum soil contaminant level of 1 mg/kg recommended in the Turkish Regulation on Control of Soil Pollution. The average contents of other HMs are below the Earth’s crust averages (mg/kg) for Ti (4500), V (90), Cr (83), Mn (1000), Fe (46,500), Co (18), Ni (58), Cu (47), Zn (83), Zr (170), and Pb (16) (Yaroshevsky 2006) and maximum soil contaminant levels (mg/kg) of Pb (50), Cr (100), Cu (50), Zn (150), Ni (30), and Co (20) (OG 2005). As a result of the wet process, 10–15% of U and 25–30% of Th are concentrated in PG, and the other major part is in the phosphoric acid (Akfas et al. 2024). The contents of U and Th analyzed in all PG samples varied from 1.1 to 46.9 mg/kg with an average of 4.9 mg/kg and 1.1 to 9.1 mg/kg with an average of 4.2 mg/kg, respectively. The average content of U is approximately two times higher than the Earth’s crust average of 2.5 mg/kg, while the average content of Th is approximately three times smaller than the crustal average of 13 mg/kg (Yaroshevsky 2006).

From Table 4, the highest contents of Ti, Cr, Mn, Fe, Ni, Cu, Zn, Zr, Cd, Pb, and U were analyzed in PG samples supplied from the factory-coded PFF4. The highest contents, V, Co, and Th, were analyzed in PG samples supplied from the factory-coded PFF3 and PFF1, respectively. According to the average contents, HMs and NORMs in the PG samples from PFFs were ranked as follows: U < Th < V < Pb < Cd < Ni < Mn < Co < Cu < Cr < Zn < Ti < Fe (PFF1); U < Cr < Cu < Cd < Zn < Ni < Pb < V < Th < Zr < Mn < Ti < Fe (PFF2); Zn < Ni < Th < Pb < Cu < Cr < U < V < Mn < Ti < Fe (PFF3); and Th < U < V < Cd < Zr < Pb < Cu < Cr < Ni < Mn < Zn < Ti < Fe (PFF4). The contents of HMs and NORMs in the studied PG samples are consistent with the value ranges analyzed in PGs from different countries given in the last column of Table 4.

**Fig. 5:**
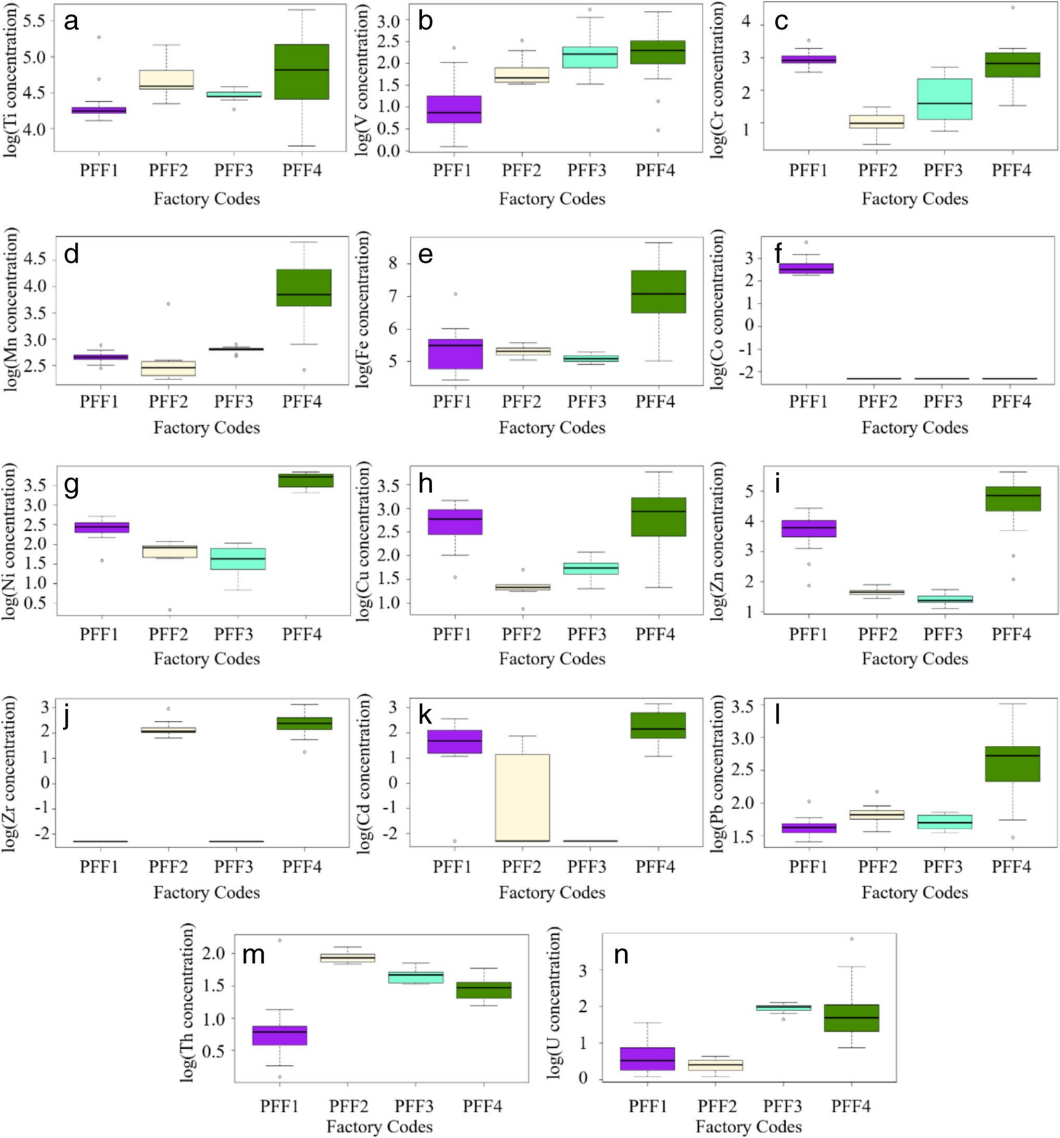
The logarithmic transformed distributions of HM and NORMs in different factories. Distributions are presented for **a** Ti, **b** V, **c** Cr, **d** Mn, **e** Fe, **f** Co, **g** Ni, **h** Cu, **i** Zn, **j** Zr, **k** Cd, **l** Pb, **m** Th, and **n** U

Figure 5 presents the distributions of logarithmically transformed HM and NORM concentrations across different factories. Statistical analysis revealed that Ti and V concentrations in PFF1 were significantly lower (*p* < 0.05) compared to PFF2, PFF3, and PFF4, which showed no significant differences among each other. For Cr concentrations, PFF1 and PFF4 were similar (*p* > 0.05), as were PFF2 and PFF3. However, Cr levels between the groups (PFF1 and PFF4 vs. PFF2 and PFF3) differed significantly (*p* < 0.05).

Mn, Ni, and Zn concentrations were found to be significantly different across all factories (*p* < 0.05). Fe concentrations were similar in PFF1, PFF2, and PFF3 (*p* > 0.05), but these factories showed significantly different Fe levels compared to PFF4 (*p* < 0.05). For Co, PFF2, PFF3, and PFF4 had similar concentrations (*p* > 0.05), while PFF1 exhibited significantly different levels (*p* < 0.05).

The analysis indicated that Ni, Cd, Pb, and Th concentrations in PFF2 and PFF3 were similar (*p* > 0.05), whereas PFF1 and PFF4 had significantly different concentrations from both PFF2 and PFF3, as well as from each other (*p* < 0.05). Zr concentrations were found to be similar between PFF1 and PFF3 and PFF2 and PFF4 (*p* > 0.05) but significantly different between these two groups (*p* < 0.05). Lastly, U concentrations were similar in PFF1 and PFF2 and PFF3 and PFF4 (*p* > 0.05) yet differed significantly between these pairs (*p* < 0.05).

**Fig. 6:**
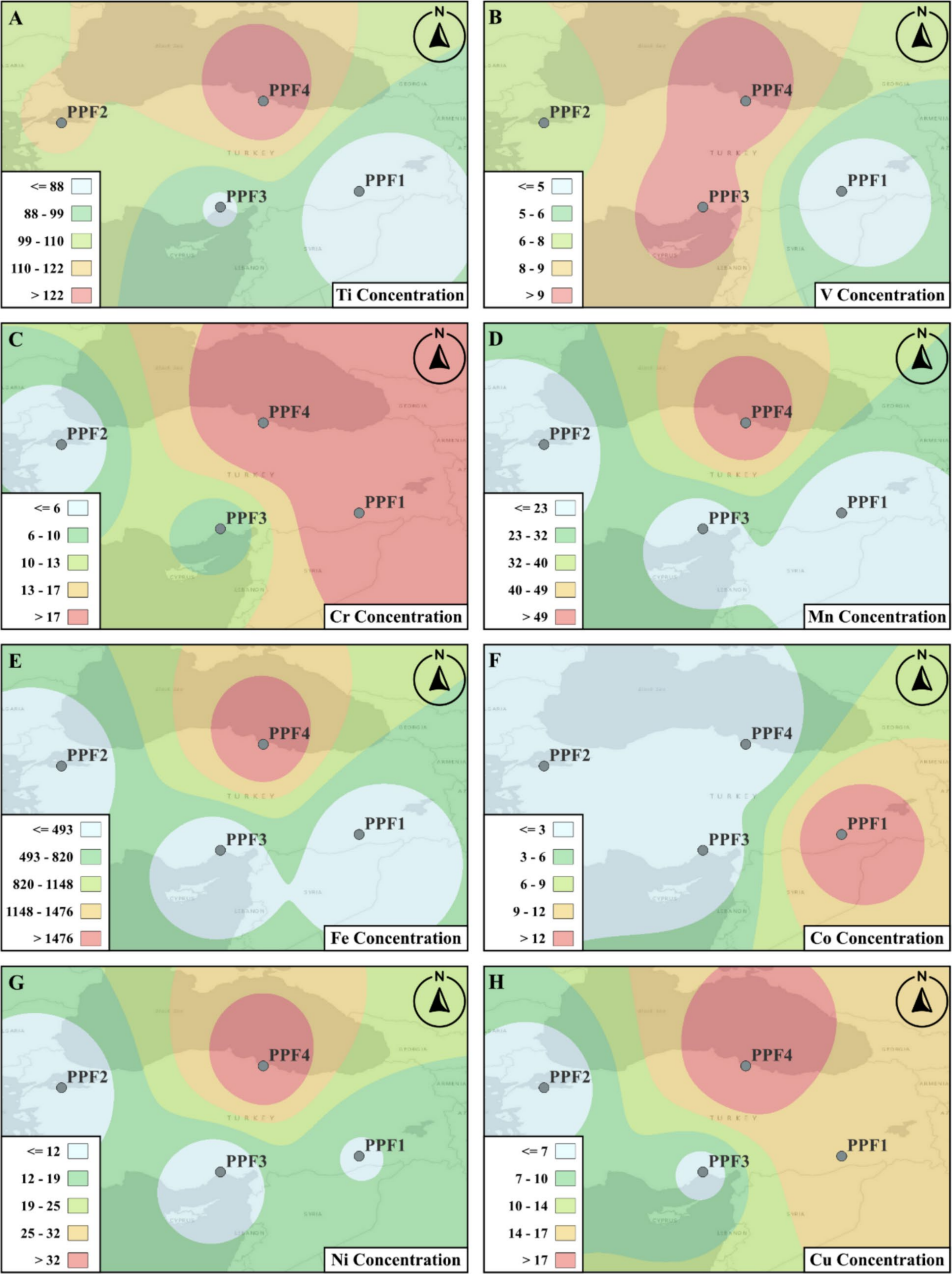
The logarithmic transformed distributions of HM and NORMs. Spatial distributions are presented for **A** Ti, **B** V, **C** Cr, **D** Mn, **E** Fe, **F** Co, **G** Ni, and **H** Cu

**Fig. 7:**
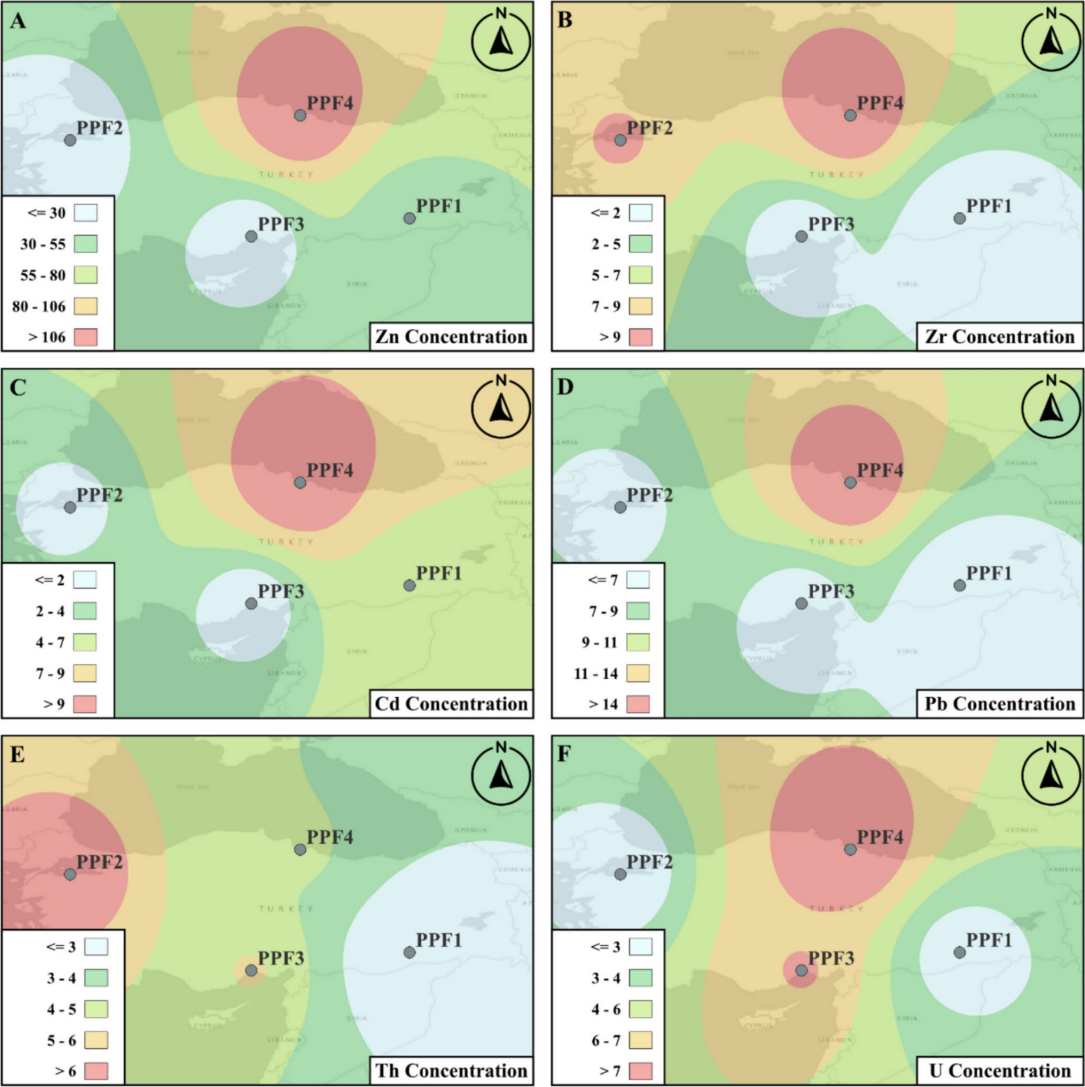
The logarithmic transformed distributions of HM and NORMs. Spatial distributions are presented for **A** Zn, **B** Zr, **C** Cd, **D** Pb, **E** Th, and **F** U

The spatial distribution analysis of HM and NORMs for the factories was conducted to present the variation in concentrations throughout collection points, which are shown in Figs. 6 and 7.

**Fig. 8:**
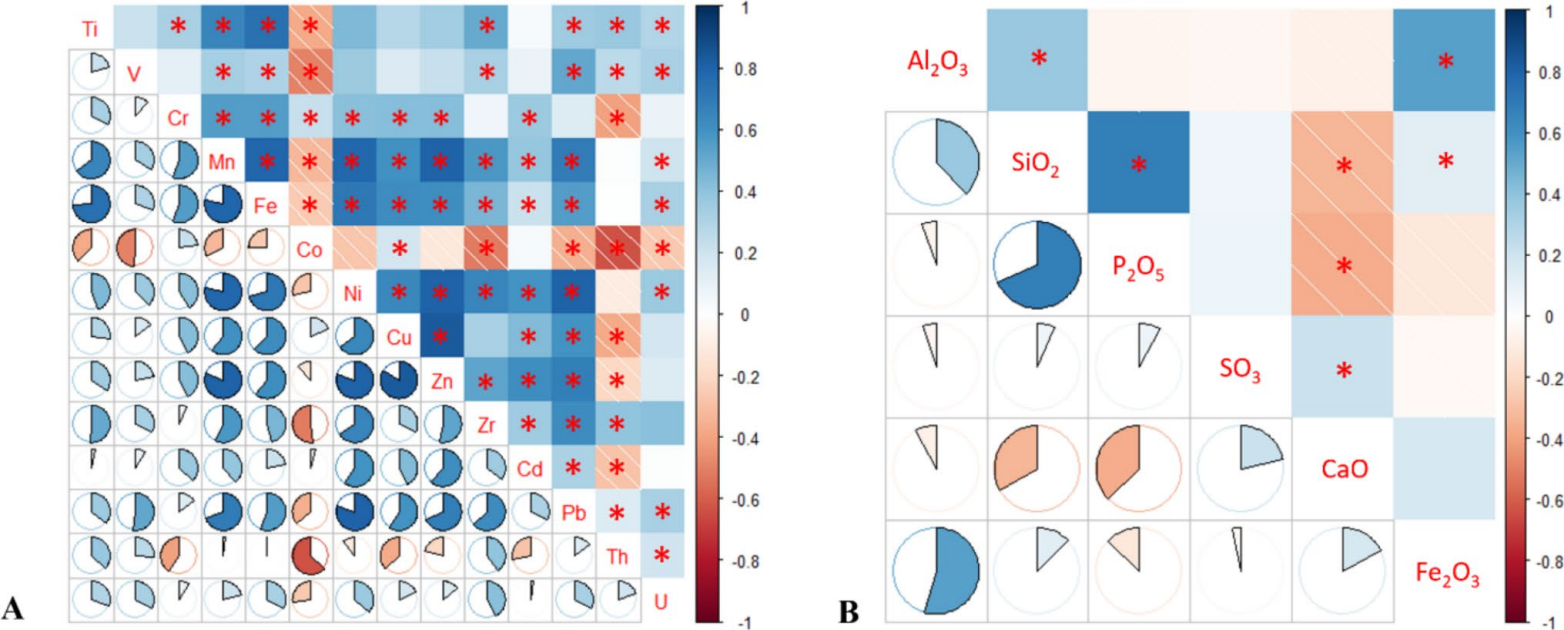
Correlation plot for HM and NORMs. **A** HM and NORMs. **B** Major and minor oxides (the asterisk “*” denotes statistically significant correlation)

In addition to these analyses, Pearson correlation analysis (Fig. 8) was conducted to examine pairwise correlations among HMs and NORMs, as well as major and minor oxides in the samples. Figure 8 highlights several significant positive and negative correlations between HMs, NORMs, and oxides.

## Conclusions

As mentioned before, huge amounts of phosphogypsum produced as waste in the phosphate industry are usually slurred with water and then pumped to a sedimentation/disposal area near the industrial fertilizer plant. The exposed piles discharge toxic pollutants (heavy metals, radionuclides, etc.) into water and soil due to the leaching of chemical species. Thus, phosphogypsum stockpiles constitute a serious environmental problem that is a potential hazard for groundwater and human health contamination. However, phosphogypsum has been used as raw material in various industrial applications such as agriculture (fertilization, land reclamation, improvement of saline and sodium soils) and construction (production of Portland cement and bricks). Therefore, the alternative decision to allow the phosphogypsum stockpile to remain in situ with a reasonable improvement or to apply phosphogypsum in industrial applications requires a comprehensive assessment of the chemical composition of phosphogypsum and the characterization of its properties. In this study, the distribution of major-minor oxides, heavy metals, and natural radionuclides in the phosphogypsum stockpiles of existing phosphate fertilizer factories in Türkiye was determined for the first time. The following main conclusions were obtained from the study: (i) the average values of SO_3_, CaO, and P_2_O_5_ were found to be significantly larger than the Earth’s crustal averages; (ii) it was observed that the Cd contents of the phosphogypsum samples were greater than the maximum soil contaminant level and Earth’s crustal average. The highest contents of Cd were analyzed in the phosphogypsum samples obtained from the factory in Samsun province; (iii) natural radioactive elements Th and U were analyzed in phosphogypsum samples. Radioactive potassium was not observed in phosphogypsum samples. The average content of U was found to be about two times greater than the average in the Earth’s crustal average.

As a result, the data obtained in this study are guiding information that can contribute to the safer reuse and waste management of these huge phosphogypsum wastes. It is recommended that the heavy metal and radionuclide contents of phosphogypsum wastes be taken into account in the evaluation and that phosphogypsum with lower contents be preferred for waste storage.

## Supplementary Information

Below is the link to the electronic supplementary material.

Supplementary file1 (DOCX 31 KB)

## Data Availability

Not applicable.
