# Supplementary material for: Dispersion of oxides, heavy metals, and natural radionuclides in phosphogypsum stockpiles of the phosphate industries in Türkiye
Source: Environ Sci Pollut Res Int. 2025 Mar 5;32(12):7692–704. doi: 10.1007/s11356-025-36180-2 (PMC11950078; doi:10.1007/s11356-025-36180-2)
Supplement: Supplementary file 1 — Supplementary file1 (DOCX 31 KB) [file 11356_2025_36180_MOESM1_ESM.docx]

Supplementary materials

**Title:** Dispersion of major-minor oxides, heavy metals and natural radionuclides in phosphogypsum stockpiles of the phosphate industries in Türkiye

**Authors:** Ş. Turhan, E.M Altuner, A. Ayata, F. Gezer, A. Hançerlioğulları, A. Kurnaz, M. Karataşlı

Table S1. Specifications of Features of EDXRF spectrometer

| **Excitation** | • X-ray tube with thick binary Pd/Co alloy anode with air-cooling • Maximum power of 50 W • Maximum of 60 kV • Adaptive excitation system with optimized filters • Bandpass filter for improved performance for elements in the range K-Mn |
| --- | --- |
| **Detection** | • Silicon drift detector with Peltier cooling. It has delivered high resolution with low spectral • Large detector area (30 mm^2^, active area 20 mm^2^) • Spectral resolution (FWHM) ≤ 155 eV  • Input count rate up to 1,000,000 counts/s • Detector filter changer for active pile-up reduction  • Application range extended to multilayer analysis, up to 8 layers and up to 55 elements  • No required calibration of certified reference samples. The user simply supplies relevant initial samples to build the database; the software examines the measured spectra, and creates a fingerprint for each sample. Going forward, every newly introduced sample can be swiftly fingerprinted, and compared to the rest of the database.  • Easy validation. Instead of validating each calibration for each element in each matrix, users simply check all samples of the database against each other and see the outcome. |
